# MicroRNA-550a is associated with muscle system conferring poorer survival for esophageal cancer

**DOI:** 10.1042/BSR20181173

**Published:** 2019-05-31

**Authors:** Housong Hong, Taisheng Liu, Huazhen Wu, Jinye Zhang, Xiaoshun Shi, Xiaobing Le, Allen M. Chen, Haiyun Mo, Qianqian Huang, Huaping Zhou, Xuguang Rao

**Affiliations:** 1 Department of Thoracic Surgery, Affiliated Cancer Hospital and Institute of Guangzhou Medical University, Guangzhou, Guangdong, China; 2 Department of Radiology, The Sixth Affiliated Hospital of Guangzhou Medical University, Guangdong, China; 3 Institute of Life Science, Nanchang University, Nanchang, China; 4 Department of Mathematics, University of California, Berkeley, CA, United States of America; 5 Department of Public Health, Guangzhou Medical University, Guangzhou, Guangdong, China; 6 Department of Hospital Infection Control, Affiliated Cancer Hospital and Institute of Guangzhou Medical University, Guangzhou 510182, Guangdong, China

**Keywords:** esophageal carcinoma, miR-550a, prognosis, TCGA

## Abstract

**Background** Esophageal cancer (ESCA) is one of the most common cancers in the digestive tract. Approximately 300000 people on an average die of ESCA per year worldwide. The determination of key microRNAs for the prognosis of ESCA is of indispensable significance in the clinical treatment. **Methods** The differentially expressed microRNAs were screened by analyzing The Cancer Genome Atlas (TCGA) database. By using the survival data of the database, we analyzed correlation between patients’ survival time and miR-550a expression levels. Differential expression analysis and gene set enrichment analysis were performed using the targeted data. **Results** It was found that patients with high miR-550a expression levels had shorter survival time. Data mining and signal pathway enrichment analysis of TCGA database showed that abnormal miR-550a expressions affected the recurrence of tumors by the muscle system regulation. **Conclusions** Through the proposed investigation, miR-550a is found to be a potential biomarker as well as non-coding therapeutic target for esophagus cancer. These results suggest that miR-550a may serve as a therapeutic target and predictor for ESCA survival.

## Introduction

Esophageal carcinoma (EC) is one of the most common malignant tumors in digestive system, ranking first in estimated new cancer cases and deaths for both men and women—untranslated region (UTR) of their target mRNAs to post-transcriptionally regulated gene expression [1,2]. Emerging evidence supports that the expression aberration of miRNAs is associated with cancer development and it serves as a potential biomarker in esophageal cancer (ESCA) [3]. For example, a group of differentially expressed miRNAs in patients with esophageal squamous cell carcinoma can effectively predict the efficacy of neoadjuvant chemoradiotherapy for esophageal squamous cell carcinoma [4]. Reports showed that miR-508 negatively regulates factors of the PI3K/Akt pathway, maintaining the phosphorylation of phosphoinositide [5]. It also activates Akt, which increases cell proliferation, anti-apoptosis, cell invasion, and ultimately leads to the formation of cancer [5].

The Cancer Genome Atlas (TCGA) project was initiated by the U.S. Government, planning through the application of genome analysis, especially through the application of large-scale genome sequencing, to print out genomic variation map of all human cancers. By systematic analysis of all oncogenic and tumor suppressor genes of small variation, the mechanism of the cancer occurrence and development may have a better understanding, which finally can draw the outline of the new ‘cancer prevention strategies’ (https://gdc.nci.nih.gov/). With the aid of next generation sequencing, miRNA expression profiles are available to be provided from the open database.

We performed a differential analysis to filter out potential miRNAs that are associated with esophageal cancer (ESCA). The survival analysis indicates that miR-550a affects the survival of ESCA patients. Subsequent pathway analysis and biomolecular validation showed that miR-550a might target the genes associated with muscle system. This pipeline may help to identify new miRNAs that influence ESCA outcomes.

## Materials and methods

### Data mining of differentially expressed miRNAs in ESCA and normal tissues

The miRNA expression and survival data of the corresponding 184 ESCA patients were downloaded from TCGA database (http://cancergenome.nih.gov). The miRNA expression data include information of full tumor and matched tumors. All the tumors were from primary tumor and data from adjacent normal tissues were utilized for comparisons. The co-expressed genes of targeted miRNAs were obtained from TCGA as well. We used edgeR package [6] to analyze the differential expression of miRNAs. The |logFC| > 1 and FDR < 0.05 were used as a screening parameter and threshold for differential expression and filtering process.

### Survival analysis of differentially expressed miRNAs

We used the median expression level of each miRNA as the cut-off value to subgroup patients into low-expression (less than the median expression level) and high-expression (greater than the median expression level) classes. In this manner, the comparison is consistent between differentially expressed gene analysis and survival analysis. Kaplan–Meier method and the two-sided log-rank test were applied to compare the survival of patients with high or low expression groups by Survival R package [7]. The *P*-value <0.05 was considered as statistically significant.

### Predictions of miRNA targets and functional analyses

Since hsa-mir-550a-3 was the unique differentially expressed miRNA associated with overall survival of ESCA, target genes of hsa-mir-550a-3 were predicted by using the miRDB web-based tool [8]. The Pearson correlation coefficient and two-sided Z-test were used to analyze the correlation between hsa-mir-550a-3 and predicted target genes. Genes with negative correlations were filtered out as potential target genes. Then the functional Gene Ontology (GO) enrichment analysis of the putative targets was performed as previously reported [9]. For each correlated and GO term, *P*-value less than 0.05 was considered as statistically significant enrichment.

### Plasmid constructs

The plasmid expressing pEZX-MR03-miR-550a-3 was purchased from GeneCopoeia (HmiR1064-MR03, Rockville, MD, U.S.A.). ESCA cells EC109 were transiently transfected with virus produced by the plasmid according to the manufacturer’s instructions. EC109 (purchased from the State Key Laboratory of Molecular Oncology, Chinese Academy of Medical Sciences) was chosen for the experiments since the citric acid prevents EC109 cell growth by inhibiting cell proliferation and inducing apoptosis, which offers guidance for its application in ESCA treatment [10]. In this regard, our bioinformatics predictions could be properly validated. The expression level of miR-550a-3 and the predicted genes were detected by qPCR at 48 h post transfection.

### RNA extraction and real-time quantitative RT-PCR assay

Real-time PCR was performed as the manual of the qRT-PCR Detection Kit (GeneCopoeia, All-in-One™ miRNA). In brief, the miRNA first-strand synthesis kit was used to perform the first-strand synthesis. The qRT-PCR analysis was performed with a specific primer (Supplementary Table S1) to hsa-mir-550a-3 (GeneCopoeia, Hmirqp0648) and the genes that predicted (Supplementary Tables S2 and S3). Since the reference genes for non-coding RNA research are U6, U48 and U1 rather than GAPDH and actin, we used snRNA U48 (GeneCopoeia, HmirQP9021) as the endogenous control without loss of generalities. SYBR-Green PCR was then performed in triplicate as well as negative controls. The 2^−ΔΔ*C*^_t_ method was used to analyze the relative expression levels [11].

### Statistical analysis

The Student’s *t* test was performed to compare the differences between the subgroups, and *P*-value <0.05 was considered to be statistically significant.

## Results

### Differentially expressed miRNAs in ESCA

Analysis of the miRNA expression profiles in ESCA compared with normal tissues identified a total of 143 differentially expressed miRNAs (Supplementary Table S4), which were used for subsequent survival analyses. Compared with normal tissues, 82 miRNAs were overexpressed. While 61 miRNAs were underexpressed in the ESCA tissues.

### The association of miRNAs and overall survival time

**Figure 1:**
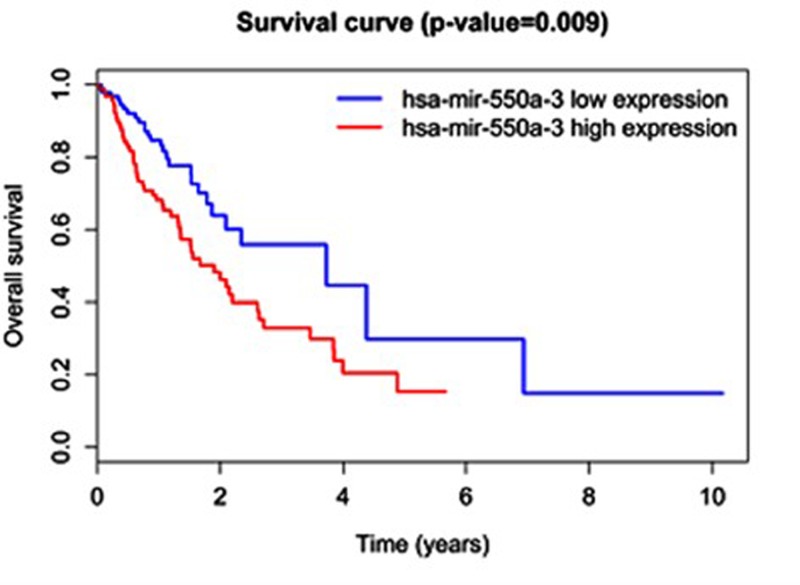
Survival curves for hsa-mir-550a-3 expression in ESCA The differences between the high-risk and low-risk groups were determined by the two-sided log-rank test.

In order to identify the differentially expressed miRNAs that were associated with overall survival of ESCA patients, log-rank test was used to determine statistically significant miRNAs. Using *P*-value 0.01 as the threshold, only one miRNA (hsa-mir-550a-3) was identified to be associated with overall survival of ESCA. The hsa-mir-550a-3 miRNA was overexpressed in ESCA comparing with normal tissues (logFC = 1.3, FDR = 0.011), and patients with higher expression of hsa-mir-550a-3 had shorter survival periods. As shown in Figure 1, high-expression group was correlated with poor prognosis of overall survival (log-rank test, *P*=0.009). Five-year overall survival rate was 29.8% (95% CI: 11.5–77.4%) and 15.4% (95% CI: 6.7–35.2%) respectively for the low-expression and high-expression groups.

### Target genes of hsa-mir-550a-3

**Figure 2:**
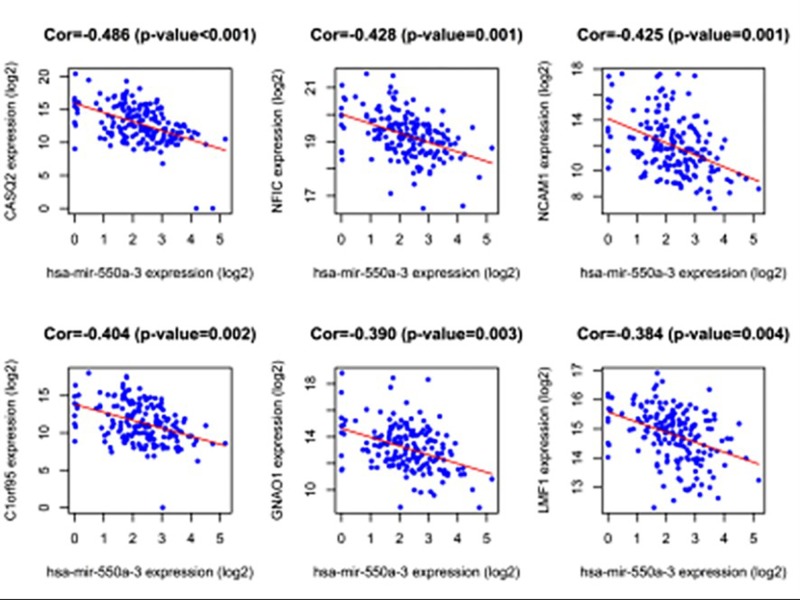
Scatter plots that indicate the expression levels of hsa-mir-550a-3 and the six genes with top correlation coefficient

Via the use of the miRDB website, the putative targets of miRNAs were predicted (Supplementary Tables S2 and S3). Since tools for predicting target genes may cause high false positive and false negative rates [12], we used co-expression method to validate the targets in ESCA. microRNA can degrade and inhibit target gene mRNAs by the specific base pairing, which negatively regulates the expression of target genes [13]. We found that 29 genes negatively correlated with hsa-mir-550a-3 (Table 1). The scatter plots for six genes with top correlation coefficient were shown in Figure 2.

**Table 1:** Targets negatively correlate with hsa-mir-550a-3

miRNA	Target gene	Correlation	*P*-value
hsa-mir-550a-3	*CASQ2*	−0.486	<0.001^†^
	*NFIC*	−0.428	0.001^†^
	*NCAM1*	−0.425	0.001^†^
	*C1orf95*	−0.404	0.002^†^
	*GNAO1*	−0.390	0.003^†^
	*LMF1*	−0.384	0.004^†^
	*ZDHHC15*	−0.372	0.005^†^
	*DTNA*	−0.371	0.005^†^
	*SLC9A9*	−0.367	0.006^†^
	*ATP1A2*	−0.358	0.007^†^
	*SPSB1*	−0.353	0.008^†^
	*THBS3*	−0.338	0.011*
	*HECA*	−0.334	0.012*
	*COL19A1*	−0.331	0.013*
	*FGF1*	−0.322	0.016*
	*ST6GALNAC6*	−0.320	0.017*
	*MSRB3*	−0.319	0.017*
	*MAB21L1*	−0.315	0.019*
	*TSHZ3*	−0.309	0.021*
	*SESN3*	−0.301	0.025*
	*TRIM9*	−0.298	0.027*
	*SCARA3*	−0.295	0.029*
	*ADCYAP1*	−0.294	0.029*
	*CRTAC1*	−0.292	0.030*
	*PDE1A*	−0.291	0.031*
	*BCL2L2*	−0.285	0.035*
	*ITPRIP*	−0.282	0.037*
	*CDIP1*	−0.282	0.037*
	*RGS9BP*	−0.274	0.044*

*P<0.05. ^†^*P*<0.01.

### mir-550a-3 negatively regulates expression levels of genes that associate muscle system in EC109 cells

**Figure 3:**
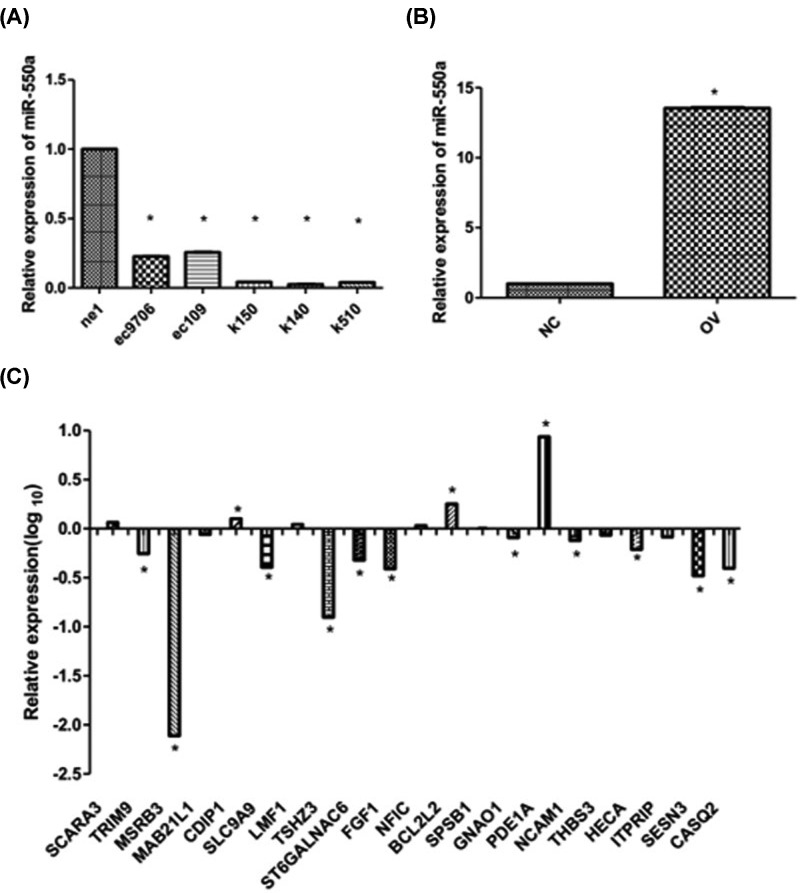
The regulation of muscle system associated genes by miR-550a-3 (**A**) mir-550a-3 is lowly expressed in ESCA cells comparing with normal esophageal cells (**B**) mir-550a-3 is ectopically expressed in EC109 cells (**C**) 77.8% predicted genes were negatively regulated by mir-550a-3 (*P-value <0.05).

Using qPCR, we determined whether miR-550a-3 regulated the expression of the above-mentioned 29 target genes, which were putative targets of miR-550a-3 based on bioinformatics analysis. First, the miR-550a-3 was detected in ESCA cell lines (Figure 3A). Then, we constructed virus to stably ectopically expressed miR-550a-3 in EC109 cells and the expression level was detected by qRT-PCR (Figure 3B). Of 29 genes, 21 genes were detected. As shown in Figure 3C, 77.8% of the predicted genes were decreased in EC109 transfected with miR-550a-3 demonstrating that miR-550a-3 negatively regulated the expression levels of these genes (**P<0.01).

### Functional roles of the identified prognostic miRNA

**Figure 4:**
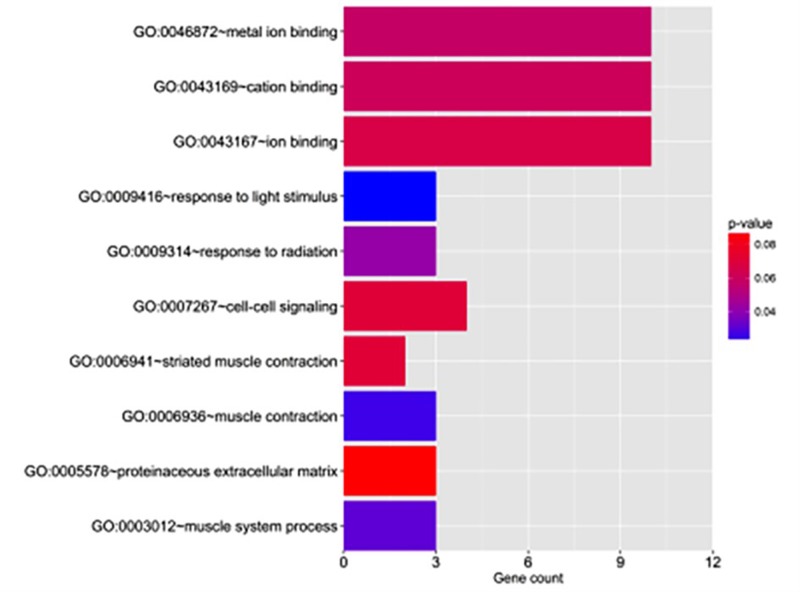
Numbers of significant target genes assigned to enriched GO terms The enriched GO terms show that gene sets involved in response to light stimulus, muscle contraction, muscle system process, and response to radiation (*P*-value <0.05).

GO enrichment was performed using the online software DAVID. The GO terms with *P*-value <0.05 are listed in Table 2. Interestingly, targets of hsa-mir-550a-3 were found to be relevant for ion binding and muscle system process (Figure 4). In ESCA, muscle depletion was related to morbidity and mortality [14]. Our findings suggested that hsa-mir-550a-3 might play roles in ESCA through muscle system.

**Table 2:** GO enrichment result for miR-550a negative correlated genes

GO term	Term name	Count	%	*P-v*alue	Genes
MF	GO:0046872∼metal ion binding	10	35.7	0.06	*ZDHHC15, SLC9A9, TSHZ3, CRTAC1, TRIM9, ATP1A2, THBS3, CASQ2, DTNA, MSRB3*
MF	GO:0043169∼cation binding	10	35.7	0.06	*ZDHHC15, SLC9A9, TSHZ3, CRTAC1, TRIM9, ATP1A2, THBS3, CASQ2, DTNA, MSRB3*
MF	GO:0043167∼ion binding	10	35.7	0.07	*ZDHHC15, SLC9A9, TSHZ3, CRTAC1, TRIM9, ATP1A2, THBS3, CASQ2, DTNA, MSRB3*
BP	GO:0009416∼**response to light stimulus**	3	10.7	0.02*	*ATP1A2, SCARA3, RGS9BP*
BP	GO:0009314∼**response to radiation**	3	10.7	0.04*	*ATP1A2, SCARA3, RGS9BP*
BP	GO:0007267∼cell-cell signaling	4	14.3	0.07	*ATP1A2, FGF1, DTNA, ADCYAP1*
BP	GO:0006941∼striated muscle contraction	2	7.14	0.07	*CASQ2, DTNA*
BP	GO:0006936∼**muscle contraction**	3	10.7	0.03*	*GNAO1, CASQ2, DTNA*
CC	GO:0005578∼proteinaceous extracellular matrix	3	10.7	0.09	*COL19A1, CRTAC1, FGF1*
BP	GO:0003012∼**muscle system process**	3	10.7	0.03*	*GNAO1, CASQ2, DTNA*

Abbreviations: BP, biological process; CC, cellular component; MF, molecular function. *P-value <0.05.

## Discussion

We downloaded 184 ESCA patients’ level 3 miRNA expression data with the corresponding survival data from the publicly available TCGA data portal website (http://cancergenome.nih.gov). Differential expression analysis was performed, which enables us to identify a total of 143 differentially expressed miRNAs. We further used these differentially expressed miRNAs for subsequent survival analyses. As shown in Figure 1, high-expression group was correlated with poor prognosis for overall survival (log-rank test, *P*=0.009). Five-year overall survival rate was 29.8% (95% CI: 11.5–77.4%) and 15.4% (95% CI: 6.7–35.2%) for the low- and high-expression group respectively. Finally, with *P*-value of 0.01 as the threshold, only miR-550a is indicated to be associated with overall survival of ESCA in our model.

MicroRNA (miRNA) is a class of endogenous small RNAs with a length of approximately 20–24 nucleotides. It has important regulatory roles in the cell mechanism. By bioinformatics analysis, miR-550a is believed to be a regulator of carcinogenesis by regulating muscle associated gene expression. Tian et al. [15] demonstrated that miR-550a was significantly up-regulated in hepatocellular carcinoma (HCC) and promoted HCC cell migration and invasion by targeting *CPEB4* gene. Furthermore, Wang et al. [16] reported that miR-550a-5p/RNF43/Wnt/β-catenin signaling pathway is responsible for colorectal cancer metastasis *in vitro* and *in vivo*. However, miR-550a gradually decreased during breast cancer initiation and progression and miR-550a-3p exerts its tumor-suppressor role by directly repressing ERK1 and ERK2, which reduce progression and metastasis of breast cancer cell [17]. The role of hsa-mir-550a-3 in the proliferation, migration, and invasion of esophageal carcinoma cell was not very clear. Although the roles of miR-550a and downstream effectors in ESCA are still unknown, evidence has suggested that miR-550a could govern carcinogenesis process by regulating downstream genes’ functions.

Our experimental data supported that over 71.4% of the predicted miR-550a target genes were down-regulated when miR-550a was overexpressed in ESCA cell lines such as EC109. Being consistent with the prognostic role of miR-550a in ESCA, some down-regulated genes are previously reported as tumor suppressor genes. For example, the expression of *TRIM9* in non-small cell lung cancer cell lines was significantly lower in HBE cells [18]. While in breast cancer, the expression of *TRIM9* was inhibited due to the methylation of *TRIM9* promoter and *TRIM9* methylation of DNA could be used as a tumor marker of breast cancer [19]. Recently, studies have shown that *SLC9A9* is down-regulated in hormone refractory prostate cancer compared with hormone-sensitive prostate cancer [20]. The functional GO enrichment analysis of the putative targets of miR-550a was performed, the findings suggest that miR-550a may play its roles in ESCA through regulating muscle system. For the enrichment pathways that are associated with muscle system, there are three genes, say, *GNAO1, CASQ2* and *DTNA*, which are overlapped with muscle system pathway. Since the identified genes are limited, we could not get a more general definite conclusion. However, the down-regulation of *GNAO1* was found to increase cell proliferation, indicating that gnao1 is a potential biomarker for HCC [21]. Our GO enrichment analysis is consistent with that result. In addition, Wang et al. [22] reported that *HECA* homologous protein can inhibit cell proliferation to a certain extent through blocking the cell cycle and it may serve as a potential molecular marker for the diagnosis of HCC. Therefore, the miR-550a might inhibit tumor suppressor’s network to affect the growth and metastasis process of ESCA.

In recent years, the association between muscle and cancer has been studied, and there is currently no effective treatment for cancer related myasthenia gravis. Studies have shown that the disordered structure of muscles has a negative impact on the survival of cancer patients. Guttridge et al. pointed out that cachexia is associated with the damage of skeletal muscle cells and the induction of muscle stem cell proliferation [23]. The overexpression of muscle stem cell factor *PAX7* prevents cell differentiation and promotes cancer induced muscle wasting. In addition, Guise et al. showed that the skeletal muscle weakness caused by advanced cancers severely reduce the quality of life of patients and increase the risk of fracture [24]. Chang et al. [25] used RT-qPCR method to detect miR-655 expression in ESCA cells and specimens. The miR-375 level in clinical esophageal carcinoma was measured using RT-qPCR, revealing that the miR-375 level was significantly decreased in tumor tissues [26]. In this study, we found that the ESCA prognosis-related miR-550a can negatively regulate the expression of muscle related genes, suggesting that the miRNA may take part in cancer-associated muscle weakness. Targeting miR-550a in ESCA patient is a potential solution for the restoration of muscle function, improving the quality of life in cancer patients with cachexia. One limitation of our research is that, for now, the bioinformatics findings lack further biological assays to confirm and validate. For instance, using qPCR to check the miR-550a levels in ESCA cells *in vitro* with various conditions. While at least, current study provides insights for pursuing the idea with experiments, which are expected in the near future.

## Conclusion

We present bioinformatics evidence indicating that miR-550a may affect the survival of ESCA patients by targeting muscle system associated genes. High miR-550a expression was correlated with shorter survival period. In addition, some target genes that were previously reported as tumor suppressors, are found to be negatively regulated by miR-550a. These results indicate that miR-550a may serve as a therapeutic target as well as predictor for ESCA survival. But a better understanding of miR-550a function during carcinogenesis needs to be further validated. Certainly, further analyses and clinical experiments are needed to confirm current conclusions.

## Supporting information

**Supplemental Table S1:** 

## Abbreviations

ERK: extracellular regulated protien kinases
ESCA: esophageal cancer
FDR: false discovery rate
GO: Gene Ontology
logFC: log Fold Change
PI3K/Akt: Phosphatidylinositide 3-kinases/protien kinase B
qPCR: Quantitative polymerse chain reaction
RNF43: ring-finger protion 43
RT-PCR: Real-time polymerse chain reaction
RT-qPCR: Real-time Quantitative polymerse chain reaction
TCGA: The Cancer Genome Atlas
95%CI: 95% confidence interval
