# Supplementary material for: MicroRNA-550a is associated with muscle system conferring poorer survival for esophageal cancer
Source: Biosci Rep. 2019 May 31;39(5):BSR20181173. doi: 10.1042/BSR20181173 (PMC6542760; doi:10.1042/BSR20181173)
Supplement: Supplementary file 1 [file bsr20181173_Supp1.pdf]

H-CASQ2-F	TGCACACGGCATATTTGGGA
H-CASQ2-R	ATTAAGCCCCTCTTCTGCCC
H-NFIC-F	TGTATTCGTCCCCGCTCTG
H-NFIC-R	TCCGCTTCTCGTGCTTCTTG
H-NCAM1-F	GCAGCGAAGAAAAGACTCTGG
H-NCAM1-R	TGGATGCTCTTCAGGGTCAG
H-C1orf95(STTM)-F	CAAATCCTCACTGCCATCGT
H-C1orf95(STTM)-R	CTCCTTGTAGCCTTGGGAAATGG
H-GNAO1-F	AAGAAGACCAGTCACAGCCATT
H-GNAO1-R	AACCTCACCGATTGGGAATAGAA
H-LMF1-F	GGAAGACTGGGTACTCGGAT
H-LMF1-R	TGCCACGAAGTACACGAAGG
H-ZDHHC15-F	ACTCTGCCTGGTTATTTACCTCA
H-ZDHHC15-R	AGCGCTCCTTGTCTGTGTAG
H-DTNA-F	GAGAGTTCTGTTATGATCTCTGTTG
H-DTNA-R	TCCTAGAACATGCACACCATGA
H-ATP1A2-F	GCCGCAAATACCAAGTGGAC
H-ATP1A2-R	AAGCTGACGGCAGAACTTGA
H-SPSB1-F	GCCCCCTGTGTCCTATGATG
H-SPSB1-R	GACTTTGCCCCTGATAGCGT
H-THBS3-F	TTGCCCTAAAGTCCCCAACC
H-THBS3-R	CTGTCTGCATCTGTCTGGGT
H-SLC9A9-F	AAGAACAAGCCTTCCCGTCC
H-SLC9A9-R	GGCTGCAGAATCCTACCCTC
H-HECA-F	GTGATCGACGTGAGGATCGG
H-HECA-R	TGGTAGTCCAGGTTCCCACA
H-COL19A1-F	TCTGAGGGGCTCAAATACGA
H-COL19A1-R	AACGGTCACGGAAGTGGAAG
H-FGF1-F	GCTCACACATTTGCCCCAAG
H-FGF1-R	CCCAGGAACACAGAGCCATT
H-ST6GALNAC6-F	CTACAGCTCCAACAGTGCCA
H-ST6GALNAC6-R	CGAGAGGGCAGTGTCTTGTT
H-MSRB3-F	AATTTCCCGTCATGCCTCCC
H-MSRB3-R	TCATGGAAAGGCCAGGCAG
H-MAB21L1-F	TGATCGGCTGAACGGGATTT
H-MAB21L1-R	AGAGCTGAGTGAGGTTTGCC
H-TSHZ3-F	GACCACCTCGACCGCTATTT
H-TSHZ3-R	GTGGGTGACAGAAGCACTGA
H-SESN3-F	TGATGTCATAATGCACAGTCCT
H-SESN3-R	GGGCCCCAGAGATAACTTGC
H-TRIM9-F	TGATCCTGCCTTTGGTGTGG
H-TRIM9-R	TCAGTTCTGTTGGTGTGCGA
H-SCARA3-F	CTCAACGTCCGGAACCTCTC
H-SCARA3-R	CCTTTCACGCCCATATCTCCT

H-ADCYAP1-F	TTCTCCCCAGAATTCACGCC
H-ADCYAP1-R	TAACGAAGCCGCCCTTTTCT
H-CRTAC1-F	AACAAGAAGTGCAGTCGGGG
H-CRTAC1-R	GCGGCTGAAGAATACCCACC
H-PDE1A-F	TTCTGGAGCGCGAAAGTCAT
H-PDE1A-R	TCAAGCTCTCCCCAAAAGCA
H-BCL2L2-F	ACCCGTGAGATCCCTAACCTG
H-BCL2L2-R	CTTAAGACACAGCACTGGTGG
H-ITPRIP-F	TTGTGTCAGGTACCGCCTAA
H-ITPRIP-R	CTAGGCCCCACACAGTGAAA
H-CDIP1-F	GGGGCTGAGGGTCTTGTTCT
H-CDIP1-R	GATGGACAGGGAACAGGCA
H-RGS9BP-F	AAAGACTCGGGATGGGTGTG
H-RGS9BP-R	CAGATGTGCACTGGAAACGC

Target Rank	Target Score	Gene Symbol	Gene Description
1	99	SHISA2	shisa family member 2
2	99	MEF2A	myocyte enhancer factor 2A
3	98	BRD1	bromodomain containing 1
4	98	CDK13	cyclin-dependent kinase 13
5	97	DERA	deoxyribose-phosphate aldolase (putative)
6	95	EXPH5	exophilin 5
7	94	ZCCHC10	zinc finger, CCHC domain containing 10
8	93	ZAK	sterile alpha motif and leucine zipper containing kinase AZK
9	93	STK4	serine/threonine kinase 4
10	92	TLX1	T-cell leukemia homeobox 1
11	91	TRAK2	trafficking protein, kinesin binding 2
12	91	C1orf95	chromosome 1 open reading frame 95
13	91	PROSC	proline synthetase co-transcribed homolog (bacterial)
14	90	STK35	serine/threonine kinase 35
15	90	RPF1	ribosome production factor 1 homolog (S. cerevisiae)
16	90	MEGF8	multiple EGF-like-domains 8
17	90	FAT2	FAT atypical cadherin 2
18	89	RTN4RL1	reticulon 4 receptor-like 1
19	89	NT5DC3	5'-nucleotidase domain containing 3
20	87	ARHGAP31	Rho GTPase activating protein 31
21	87	CBFA2T2	core-binding factor, runt domain, alpha subunit 2; translocated to, 2
22	87	TMEM151B	transmembrane protein 151B
23	87	XRN1	5'-3' exoribonuclease 1
24	87	DPYSL5	dihydropyrimidinase-like 5
25	87	ZSWIM6	zinc finger, SWIM-type containing 6
26	86	LMBR1	limb development membrane protein 1
27	86	CACNB3	calcium channel, voltage-dependent, beta 3 subunit
28	86	PDGFRA	platelet-derived growth factor receptor, alpha polypeptide
29	85	TSHZ3	teashirt zinc finger homeobox 3
30	85	MTUS2	microtubule associated tumor suppressor candidate 2
31	84	CCDC3	coiled-coil domain containing 3
32	84	RAP1GAP2	RAP1 GTPase activating protein 2
33	84	APPL2	adaptor protein, phosphotyrosine interaction, PH domain and leucine zipper containing 2
34	84	ADAMTS6	ADAM metallopeptidase with thrombospondin type 1 motif, 6
35	83	RAB43	RAB43, member RAS oncogene family
36	83	CAB39	calcium binding protein 39

37	83	NGEF	neuronal guanine nucleotide exchange factor
38	83	CENPO	centromere protein O
39	83	ABCF1	ATP-binding cassette, sub-family F (GCN20), member 1
40	82	CDH5	cadherin 5, type 2 (vascular endothelium)
41	81	ARHGEF12	Rho guanine nucleotide exchange factor (GEF) 12
42	81	WTAP	Wilms tumor 1 associated protein
43	80	CCDC68	coiled-coil domain containing 68
44	80	DENND4C	DENN/MADD domain containing 4C
45	80	DAZAP2	DAZ associated protein 2
46	80	ADCY1	adenylate cyclase 1 (brain)
47	80	BCL2L2	BCL2-like 2
48	80	ATP1A2	ATPase, Na+/K+ transporting, alpha 2 polypeptide
49	79	SORCS2	sortilin-related VPS10 domain containing receptor 2
50	78	PSME1	proteasome (prosome, macropain) activator subunit 1 (PA28 alpha)
51	78	ST6GALNAC6	ST6 (alpha-N-acetyl-neuraminyl-2,3-beta-galactosyl-1,3)-N-acetylgalactosaminide alpha-2,6-sialyltransferase 6
52	78	MAN1A1	mannosidase, alpha, class 1A, member 1
53	78	ITPRIP	inositol 1,4,5-trisphosphate receptor interacting protein
54	78	IGSF21	immunoglobin superfamily, member 21
55	78	ADAMTS5	ADAM metallopeptidase with thrombospondin type 1 motif, 5
56	78	EMX2	empty spiracles homeobox 2
57	77	KRTAP20-3	keratin associated protein 20-3
58	77	MTDH	metadherin
59	77	NUDT4	nudix (nucleoside diphosphate linked moiety X)-type motif 4
60	77	ACBD3	acyl-CoA binding domain containing 3
61	76	C5orf20	chromosome 5 open reading frame 20
62	76	PPAPDC2	phosphatidic acid phosphatase type 2 domain containing 2
63	75	ADCYAP1	adenylate cyclase activating polypeptide 1 (pituitary)
64	75	STK32A	serine/threonine kinase 32A
65	74	GSG1L	GSG1-like
66	74	FBXL7	F-box and leucine-rich repeat protein 7
67	74	ESYT2	extended synaptotagmin-like protein 2
68	74	MED29	mediator complex subunit 29
69	74	LOC102725096	proline-rich protein 2-like
70	74	YAF2	YY1 associated factor 2
71	74	ALDH5A1	aldehyde dehydrogenase 5 family, member A1
72	74	C20orf112	chromosome 20 open reading frame 112
73	73	TRIM44	tripartite motif containing 44

74	73	FGF1	fibroblast growth factor 1 (acidic)
75	73	RNF43	ring finger protein 43
76	73	TBC1D10C	TBC1 domain family, member 10C
77	73	UBE2Q1	ubiquitin-conjugating enzyme E2Q family member 1
78	72	SEC62	SEC62 homolog (S. cerevisiae)
79	72	LSM12	LSM12 homolog (S. cerevisiae)
80	72	MDGA2	MAM domain containing glycosylphosphatidylinositol anchor 2
81	71	CGNL1	cingulin-like 1
82	71	RPS6KA1	ribosomal protein S6 kinase, 90kDa, polypeptide 1
83	71	SUZ12	SUZ12 polycomb repressive complex 2 subunit
84	71	C17orf102	chromosome 17 open reading frame 102
85	71	SESN3	sestrin 3
86	71	KCNMB4	potassium large conductance calcium-activated channel, subfamily M, beta member 4
87	71	SLC25A23	solute carrier family 25 (mitochondrial carrier; phosphate carrier), member 23
88	70	TIMP2	TIMP metallopeptidase inhibitor 2
89	70	TDP2	tyrosyl-DNA phosphodiesterase 2
90	69	CD22	CD22 molecule
91	69	GCNT4	glucosaminyl (N-acetyl) transferase 4, core 2
92	69	B3GAT3	beta-1,3-glucuronyltransferase 3 (glucuronosyltransferase I)
93	69	TRIM27	tripartite motif containing 27
94	69	SV2A	synaptic vesicle glycoprotein 2A
95	69	ARSB	arylsulfatase B
96	69	ZNF710	zinc finger protein 710
97	68	ZNF689	zinc finger protein 689
98	68	NDUFA6	NADH dehydrogenase (ubiquinone) 1 alpha subcomplex, 6, 14kDa
99	68	NEUROG1	neurogenin 1
100	68	MED13L	mediator complex subunit 13-like
101	68	LRRC8A	leucine rich repeat containing 8 family, member A
102	68	SAMD12	sterile alpha motif domain containing 12
103	67	STX16	syntaxin 16
104	67	PLEKHM3	pleckstrin homology domain containing, family M, member 3
105	67	SP1	Sp1 transcription factor
106	67	LUC7L3	LUC7-like 3 (S. cerevisiae)
107	67	KAT2A	K(lysine) acetyltransferase 2A
108	67	ZNRF1	zinc and ring finger 1, E3 ubiquitin protein ligase
109	66	GABRA4	gamma-aminobutyric acid (GABA) A receptor, alpha 4
110	66	COL19A1	collagen, type XIX, alpha 1
111	66	ABCG4	ATP-binding cassette, sub-family G (WHITE), member 4
112	66	HES2	hes family bHLH transcription factor 2

113	66	KIAA1549	KIAA1549
114	66	SOS1	son of sevenless homolog 1 (Drosophila)
115	66	SV2B	synaptic vesicle glycoprotein 2B
116	65	FBXO34	F-box protein 34
117	65	TMX2	thioredoxin-related transmembrane protein 2
118	65	NFIC	nuclear factor I/C (CCAAT-binding transcription factor)
119	64	IGDCC4	immunoglobulin superfamily, DCC subclass, member 4
120	64	NCAM1	neural cell adhesion molecule 1
121	64	DSTN	destrin (actin depolymerizing factor)
122	64	SOX13	SRY (sex determining region Y)-box 13
123	63	CX3CR1	chemokine (C-X3-C motif) receptor 1
124	63	USP49	ubiquitin specific peptidase 49
125	63	PDP2	pyruvate dehyrogenase phosphatase catalytic subunit 2
126	63	AIPL1	aryl hydrocarbon receptor interacting protein-like 1
127	62	SERTAD2	SERTA domain containing 2
128	62	GP1BA	glycoprotein Ib (platelet), alpha polypeptide
129	62	RIMBP2	RIMS binding protein 2
130	62	SAR1B	secretion associated, Ras related GTPase 1B
131	62	PAPOLA	poly(A) polymerase alpha
132	61	RGS9BP	regulator of G protein signaling 9 binding protein
133	61	P2RY2	purinergic receptor P2Y, G-protein coupled, 2
134	61	KRTAP4-3	keratin associated protein 4-3
135	61	DHCR24	24-dehydrocholesterol reductase
136	61	TRIM9	tripartite motif containing 9
137	61	GTDC1	glycosyltransferase-like domain containing 1
138	61	MYO1E	myosin IE
139	60	ARCN1	archain 1
140	60	KLHL12	kelch-like family member 12
141	60	MKRN1	makorin ring finger protein 1
142	60	CNST	consortin, connexin sorting protein
143	59	SCARA3	scavenger receptor class A, member 3
144	59	NUP50	nucleoporin 50kDa
145	59	BCL3	B-cell CLL/lymphoma 3
146	59	CCND2	cyclin D2
147	59	CCDC6	coiled-coil domain containing 6
148	59	TNFRSF11B	tumor necrosis factor receptor superfamily, member 11b
149	59	RNF26	ring finger protein 26
150	59	DDX17	DEAD (Asp-Glu-Ala-Asp) box helicase 17
151	59	TRIM46	tripartite motif containing 46
152	59	TMEM106A	transmembrane protein 106A
153	59	ALKBH1	alkB, alkylation repair homolog 1 (E. coli)
154	58	ITK	IL2-inducible T-cell kinase

155	58	ELAVL1	ELAV like RNA binding protein 1
156	58	ZCCHC5	zinc finger, CCHC domain containing 5
157	58	IL23R	interleukin 23 receptor
158	58	C15orf40	chromosome 15 open reading frame 40
159	57	ARL2BP	ADP-ribosylation factor-like 2 binding protein
160	57	UNG	uracil-DNA glycosylase
161	57	SLC22A23	solute carrier family 22, member 23
162	57	LRRC27	leucine rich repeat containing 27
163	57	CDC42SE2	CDC42 small effector 2
164	57	ZNF185	zinc finger protein 185 (LIM domain)
165	57	SRGAP2C	SLIT-ROBO Rho GTPase activating protein 2C
166	56	TTPA	tocopherol (alpha) transfer protein
167	56	C16orf52	chromosome 16 open reading frame 52
168	56	ARRB1	arrestin, beta 1
169	56	URM1	ubiquitin related modifier 1
170	56	RPUSD2	RNA pseudouridylate synthase domain containing 2
171	56	RGAG1	retrotransposon gag domain containing 1
172	55	BAIAP2L1	BAI1-associated protein 2-like 1
173	55	NUP93	nucleoporin 93kDa
174	55	KIAA0930	KIAA0930
175	55	TEC	tec protein tyrosine kinase
176	55	LIMD1	LIM domains containing 1
177	55	TERF2	telomeric repeat binding factor 2
178	55	FLJ44635	TPT1-like protein
179	55	THEG	theg spermatid protein
180	55	LZTS3	leucine zipper, putative tumor suppressor family member 3
181	55	KLHL6	kelch-like family member 6
182	55	HOOK3	hook microtubule-tethering protein 3
183	55	KCNE1	potassium voltage-gated channel, Isk-related family, member 1
184	54	MKI67	marker of proliferation Ki-67
185	54	TOB2	transducer of ERBB2, 2
186	54	VSIG4	V-set and immunoglobulin domain containing 4
187	54	PCDH18	protocadherin 18
188	54	LOC101927402	uncharacterized LOC101927402
189	54	LOC101059936	putative uncharacterized protein encoded by LINC00174-like
190	54	SPSB1	splA/ryanodine receptor domain and SOCS box containing 1
191	54	TM9SF4	transmembrane 9 superfamily protein member 4
192	53	AHCTF1	AT hook containing transcription factor 1
193	53	LGALS8	lectin, galactoside-binding, soluble, 8
194	53	CHURC1	churchill domain containing 1

195	53	CDIP1	cell death-inducing p53 target 1
196	53	CNOT2	CCR4-NOT transcription complex, subunit 2
197	53	ATMIN	ATM interactor
198	53	GNL1	guanine nucleotide binding protein-like 1
199	53	FBXW8	F-box and WD repeat domain containing 8
200	53	ISY1-RAB43	ISY1-RAB43 readthrough
201	53	KIAA0907	KIAA0907
202	52	ZNF417	zinc finger protein 417
203	52	LAD1	ladinin 1
204	52	THBS3	thrombospondin 3
205	52	ADPRH	ADP-ribosylarginine hydrolase
206	52	CD302	CD302 molecule
207	52	PCDH15	protocadherin-related 15
208	52	SLC9A9	solute carrier family 9, subfamily A (NHE9, cation proton antiporter 9), member 9
209	52	PLXNA2	plexin A2
210	52	PSMF1	proteasome (prosome, macropain) inhibitor subunit 1 (PI31)
211	52	GALE	UDP-galactose-4-epimerase
212	52	HAPLN4	hyaluronan and proteoglycan link protein 4
213	52	CDCA7	cell division cycle associated 7
214	52	BATF3	basic leucine zipper transcription factor, ATF-like 3
215	52	GIMAP4	GTPase, IMAP family member 4
216	52	MAPK8	mitogen-activated protein kinase 8
217	52	LY75-CD302	LY75-CD302 readthrough
218	51	RAB34	RAB34, member RAS oncogene family
219	51	LOC400863	uncharactered LOC400863
220	51	CIITA	class II, major histocompatibility complex, transactivator
221	51	ADAMTSL5	ADAMTS-like 5
222	51	GJC1	gap junction protein, gamma 1, 45kDa
223	51	TUSC5	tumor suppressor candidate 5
224	51	CA13	carbonic anhydrase XIII
225	51	OPN4	opsin 4
226	51	LMF1	lipase maturation factor 1
227	51	DHX35	DEAH (Asp-Glu-Ala-His) box polypeptide 35
228	51	ZNF93	zinc finger protein 93
229	50	UPP2	uridine phosphorylase 2
230	50	KIAA0355	KIAA0355
231	50	TMEM248	transmembrane protein 248
232	50	C17orf70	chromosome 17 open reading frame 70
233	50	ZNF229	zinc finger protein 229
234	50	SLC47A1	solute carrier family 47 (multidrug and toxin extrusion),

			member 1
235	50	ALDH8A1	aldehyde dehydrogenase 8 family, member A1
236	50	SEMA4C	sema domain, immunoglobulin domain (Ig), transmembrane domain (TM) and short cytoplasmic domain, (semaphorin) 4C

Target Rank	Target Score	Gene Symbol	Gene Description
1	100	MYH10	myosin, heavy chain 10, non-muscle
2	100	SYT4	synaptotagmin IV
3	99	HECA	headcase homolog (Drosophila)
4	99	CNIH1	cornichon family AMPA receptor auxiliary protein 1
5	97	COL11A1	collagen, type XI, alpha 1
6	97	CAPRIN1	cell cycle associated protein 1
7	96	ARID4B	AT rich interactive domain 4B (RBP1-like)
8	95	RBMXL1	RNA binding motif protein, X-linked-like 1
9	95	PCDH17	protocadherin 17
10	94	SYNCRIP	synaptotagmin binding, cytoplasmic RNA interacting protein
11	93	MSRB3	methionine sulfoxide reductase B3
12	93	MYT1L	myelin transcription factor 1-like
13	92	VASN	vasorin
14	91	RHOBTB1	Rho-related BTB domain containing 1
15	89	PTBP3	polypyrimidine tract binding protein 3
16	88	HIVEP1	human immunodeficiency virus type I enhancer binding protein 1
17	86	SMAD5	SMAD family member 5
18	85	THBD	thrombomodulin
19	85	PITPNC1	phosphatidylinositol transfer protein, cytoplasmic 1
20	84	GNS	glucosamine (N-acetyl)-6-sulfatase
21	83	UTP15	UTP15, U3 small nucleolar ribonucleoprotein, homolog (S. cerevisiae)
22	83	APPL1	adaptor protein, phosphotyrosine interaction, PH domain and leucine zipper containing 1
23	82	INA	internexin neuronal intermediate filament protein, alpha
24	82	GPR137C	G protein-coupled receptor 137C
25	81	SLC16A14	solute carrier family 16, member 14
26	80	TDP1	tyrosyl-DNA phosphodiesterase 1
27	80	PCDH8	protocadherin 8
28	79	CMAS	cytidine monophosphate N-acetylneuraminic acid synthetase
29	79	CASQ2	calsequestrin 2 (cardiac muscle)
30	77	HTR2C	5-hydroxytryptamine (serotonin) receptor 2C, G protein-coupled
31	77	SPRY2	sprouty homolog 2 (Drosophila)
32	77	ZNF763	zinc finger protein 763
33	76	CCNT1	cyclin T1
34	75	C6orf120	chromosome 6 open reading frame 120
35	75	SPACA1	sperm acrosome associated 1

36	75	ATP1A2	ATPase, Na+/K+ transporting, alpha 2 polypeptide
37	75	TRAF3	TNF receptor-associated factor 3
38	74	PCNP	PEST proteolytic signal containing nuclear protein
39	74	LOC100505498	uncharacterized LOC100505498
40	74	MAB21L1	mab-21-like 1 (C. elegans)
41	73	ZBTB8A	zinc finger and BTB domain containing 8A
42	73	KDELR2	KDEL (Lys-Asp-Glu-Leu) endoplasmic reticulum protein retention receptor 2
43	73	IL7	interleukin 7
44	73	IRAK1BP1	interleukin-1 receptor-associated kinase 1 binding protein 1
45	72	SLC25A13	solute carrier family 25 (aspartate/glutamate carrier), member 13
46	72	THAP9	THAP domain containing 9
47	72	DMXL1	Dmx-like 1
48	72	SEPT7	septin 7
49	71	C5orf54	chromosome 5 open reading frame 54
50	70	POTEF	POTE ankyrin domain family, member F
51	70	MOB4	MOB family member 4, phocein
52	70	PCMTD2	protein-L-isoaspartate (D-aspartate) O-methyltransferase domain containing 2
53	69	CRTAC1	cartilage acidic protein 1
54	69	GNG13	guanine nucleotide binding protein (G protein), gamma 13
55	68	TPRG1L	tumor protein p63 regulated 1-like
56	68	TRAK1	trafficking protein, kinesin binding 1
57	67	PAK3	p21 protein (Cdc42/Rac)-activated kinase 3
58	66	C10orf118	chromosome 10 open reading frame 118
59	66	BRD3	bromodomain containing 3
60	66	ZNF655	zinc finger protein 655
61	66	GNAI3	guanine nucleotide binding protein (G protein), alpha inhibiting activity polypeptide 3
62	66	DLD	dihydrolipoamide dehydrogenase
63	65	LINGO2	leucine rich repeat and Ig domain containing 2
64	65	MEAF6	MYST/Esa1-associated factor 6
65	64	FAM71D	family with sequence similarity 71, member D
66	64	DTNA	dystrobrevin, alpha
67	64	SEMA4F	sema domain, immunoglobulin domain (Ig), transmembrane domain (TM) and short cytoplasmic domain, (semaphorin) 4F
68	64	CCDC141	coiled-coil domain containing 141
69	63	MEF2C	myocyte enhancer factor 2C
70	63	ZDHHC15	zinc finger, DHHC-type containing 15

71	63	GNAO1	guanine nucleotide binding protein (G protein), alpha activating activity polypeptide O
72	63	PLEKHF2	pleckstrin homology domain containing, family F (with FYVE domain) member 2
73	62	BBX	bobby sox homolog (Drosophila)
74	62	HEPHL1	hephaestin-like 1
75	61	C1orf94	chromosome 1 open reading frame 94
76	61	SERINC5	serine incorporator 5
77	61	CRHBP	corticotropin releasing hormone binding protein
78	60	ZNF592	zinc finger protein 592
79	60	CPXCR1	CPX chromosome region, candidate 1
80	59	EML1	echinoderm microtubule associated protein like 1
81	59	MLANA	melan-A
82	59	PROX1	prospero homeobox 1
83	59	ZNF792	zinc finger protein 792
84	58	DHFRL1	dihydrofolate reductase-like 1
85	58	DHFR	dihydrofolate reductase
86	58	C2orf88	chromosome 2 open reading frame 88
87	58	CD47	CD47 molecule
88	58	ESAM	endothelial cell adhesion molecule
89	57	PSMD14	proteasome (prosome, macropain) 26S subunit, non-ATPase, 14
90	57	ZNF14	zinc finger protein 14
91	57	ZNF83	zinc finger protein 83
92	57	ACER3	alkaline ceramidase 3
93	57	NTRK2	neurotrophic tyrosine kinase, receptor, type 2
94	56	SLC39A9	solute carrier family 39, member 9
95	56	SMARCD2	SWI/SNF related, matrix associated, actin dependent regulator of chromatin, subfamily d, member 2
96	56	LOC101060835	HLA class II histocompatibility antigen, DQ beta 1 chain-like
97	56	DYRK2	dual-specificity tyrosine-(Y)-phosphorylation regulated kinase 2
98	54	CA8	carbonic anhydrase VIII
99	54	PTS	6-pyruvoyltetrahydropterin synthase
100	54	POTEM	POTE ankyrin domain family, member M
101	54	SLC18A2	solute carrier family 18 (vesicular monoamine transporter), member 2
102	54	CCT7	chaperonin containing TCP1, subunit 7 (eta)
103	54	INTU	inturned planar cell polarity protein
104	54	PARM1	prostate androgen-regulated mucin-like protein 1
105	53	SYCP3	synaptonemal complex protein 3
106	53	FAM110B	family with sequence similarity 110, member B
107	53	KCNAB3	potassium voltage-gated channel, shaker-related

			subfamily, beta member 3
108	53	CLEC4D	C-type lectin domain family 4, member D
109	53	ICK	intestinal cell (MAK-like) kinase
110	52	MTM1	myotubularin 1
111	52	CSNK1G2	casein kinase 1, gamma 2
112	52	TSNAX	translin-associated factor X
113	52	ZNF365	zinc finger protein 365
114	52	PDE1A	phosphodiesterase 1A, calmodulin-dependent
115	51	TNFRSF11A	tumor necrosis factor receptor superfamily, member 11a, NFKB activator
116	51	SULT2A1	sulfotransferase family, cytosolic, 2A, dehydroepiandrosterone (DHEA)-preferring, member 1
117	51	CACNB2	calcium channel, voltage-dependent, beta 2 subunit
118	51	TMEM74	transmembrane protein 74
119	51	TMEM56	transmembrane protein 56
120	51	RUNDC3B	RUN domain containing 3B
121	50	PGPEP1L	pyroglutamyl-peptidase I-like

ID	logFC	logCPM	PValue	FDR
hsa-mir-148a	-2.8885439	14.90815645	2.80E-41	1.71E-38
hsa-mir-30c-1	-1.8315059	8.674097351	1.41E-22	4.29E-20
hsa-mir-139	-2.1765843	5.940234869	8.61E-22	1.75E-19
hsa-mir-204	-4.048675	2.866729376	1.39E-21	2.12E-19
hsa-mir-30e	-1.4164697	13.28236014	7.27E-20	8.89E-18
hsa-mir-490	-4.2384068	2.244266964	3.45E-16	3.52E-14
hsa-mir-28	-1.3922893	12.2950622	1.44E-14	1.26E-12
hsa-mir-30a	-1.9305275	14.05469604	2.97E-14	2.27E-12
hsa-mir-30c-2	-1.4990384	8.736531999	1.31E-13	8.92E-12
hsa-mir-133a-1	-3.0615422	5.927598315	5.35E-13	3.27E-11
hsa-mir-135a-2	-3.1013755	0.683650412	8.74E-13	4.85E-11
hsa-mir-133a-2	-3.0343945	5.827872459	1.04E-12	5.32E-11
hsa-mir-153-2	-2.4183087	3.953782899	1.39E-12	6.55E-11
hsa-mir-664a	-1.2946798	4.876238593	3.03E-12	1.32E-10
hsa-mir-135a-1	-2.9887649	0.509795335	1.15E-11	4.68E-10
hsa-mir-26b	-1.0377537	9.340181085	1.44E-11	5.51E-10
hsa-mir-29c	-1.9750235	11.3587786	2.31E-11	8.30E-10
hsa-mir-378c	-1.6894548	3.434166279	7.14E-11	2.42E-09
hsa-mir-125a	-1.2578626	8.600296683	1.04E-10	3.34E-09
hsa-mir-145	-2.112338	11.92675431	2.27E-10	6.95E-09
hsa-mir-628	-1.2325474	2.891589061	4.45E-10	1.29E-08
hsa-mir-1468	-1.9651356	2.467556096	2.72E-09	7.56E-08
hsa-mir-153-1	-2.3679609	0.604113001	3.13E-09	7.96E-08
hsa-mir-1-2	-2.3954707	5.529438664	3.17E-08	7.74E-07
hsa-mir-202	-2.7403653	-0.03023735	5.42E-08	1.27E-06
hsa-mir-378d-1	-1.731423	-0.36538681	7.46E-08	1.69E-06
hsa-mir-365b	-1.2852575	6.631199862	9.32E-08	2.03E-06
hsa-mir-365a	-1.2820352	6.63527858	1.08E-07	2.27E-06
hsa-mir-1-1	-2.3217758	5.445909532	1.36E-07	2.77E-06
hsa-mir-93	1.23972445	13.08281897	3.76E-07	7.42E-06
hsa-mir-4664	3.96857435	0.200860694	4.30E-07	8.21E-06
hsa-mir-195	-1.2434861	4.882863632	5.94E-07	1.06E-05
hsa-mir-4652	3.7911523	1.824365523	6.09E-07	1.06E-05
hsa-mir-21	1.08107208	18.25608679	8.60E-07	1.46E-05
hsa-mir-375	-2.5667825	14.16901757	1.20E-06	1.93E-05
hsa-mir-767	7.43919565	5.832064864	1.25E-06	1.95E-05
hsa-mir-30b	-1.0847436	9.160408399	1.50E-06	2.29E-05
hsa-mir-143	-1.5243644	17.39124174	1.69E-06	2.52E-05
hsa-mir-29b-2	-1.0806028	8.699179616	1.97E-06	2.87E-05
hsa-mir-4746	1.75819286	2.34725967	2.20E-06	3.12E-05
hsa-mir-378d-2	-1.5215325	-0.1867516	3.83E-06	5.20E-05
hsa-mir-642a	-1.449778	1.330896677	5.99E-06	7.79E-05
hsa-mir-106b	1.05761777	9.308860538	6.42E-06	8.17E-05

hsa-mir-133b	-2.2432356	3.412148328	7.60E-06	9.48E-05
hsa-mir-224	3.03058769	6.957449508	7.86E-06	9.61E-05
hsa-mir-497	-1.0721476	3.82475651	8.11E-06	9.71E-05
hsa-mir-1258	-1.8839347	0.135759282	1.16E-05	0.000131
hsa-mir-383	-2.5361516	-0.1064339	1.22E-05	0.000135
hsa-mir-105-2	6.56799363	4.986397082	1.28E-05	0.000139
hsa-mir-151b	-1.1687581	0.4619329	2.17E-05	0.000229
hsa-mir-105-1	5.97630333	4.991018002	2.79E-05	0.000289
hsa-mir-301b	2.07400302	1.026950728	2.95E-05	0.0003
hsa-mir-335	1.49329484	6.727788674	3.28E-05	0.000329
hsa-mir-218-1	-1.3038312	4.050750587	3.76E-05	0.000371
hsa-mir-937	2.25608157	1.561622015	4.19E-05	0.000406
hsa-mir-6874	-1.5737459	-0.52981875	4.71E-05	0.000447
hsa-mir-664b	-1.0040783	1.96508031	4.76E-05	0.000447
hsa-mir-135b	1.99643763	5.829752358	5.31E-05	0.000477
hsa-mir-1304	2.28366572	1.20746461	5.81E-05	0.000514
hsa-mir-129-1	-1.9665882	2.119840648	6.84E-05	0.000597
hsa-mir-18a	1.39656441	5.631237411	7.14E-05	0.000615
hsa-mir-218-2	-1.278767	4.006457185	7.56E-05	0.000634
hsa-mir-877	1.57409772	2.154286236	7.59E-05	0.000634
hsa-mir-6507	-2.077333	-0.02620453	7.68E-05	0.000634
hsa-mir-421	1.18748345	2.365084535	0.000101	0.000825
hsa-mir-129-2	-1.7772303	2.117296216	0.000105	0.000847
hsa-mir-100	-1.3528267	11.37203337	0.00011	0.000861
hsa-mir-181b-1	1.04834666	7.283750694	0.000151	0.001137
hsa-mir-483	4.06333781	3.573878705	0.000193	0.001437
hsa-mir-125b-1	-1.0510361	7.625443718	0.000223	0.001639
hsa-mir-301a	1.02809954	3.681919441	0.000234	0.0017
hsa-mir-615	1.93309945	1.677055492	0.000261	0.001855
hsa-mir-125b-2	-1.0364362	7.717940486	0.000316	0.002153
hsa-mir-338	-1.1517754	10.01658666	0.000407	0.002672
hsa-mir-6891	3.75503014	-0.39648027	0.000413	0.002687
hsa-let-7c	-1.2404872	10.79741284	0.000445	0.002864
hsa-mir-1262	-1.0376091	0.60912459	0.000472	0.003006
hsa-mir-4521	-1.45572	0.254550876	0.000488	0.003075
hsa-mir-452	1.88247139	7.218991468	0.000511	0.003183
hsa-mir-3662	3.24204787	2.274207192	0.000548	0.003348
hsa-mir-455	1.16049491	7.638553251	0.000557	0.003372
hsa-mir-363	-1.2034124	3.363261362	0.000633	0.003792
hsa-mir-676	-1.3337122	0.221453882	0.000675	0.003926
hsa-mir-1180	1.11227665	4.162165374	0.00078	0.004496
hsa-mir-548f-1	4.08174438	0.89827341	0.000826	0.004673
hsa-mir-372	10.8368435	7.415889604	0.000847	0.004705
hsa-mir-550a-1	1.30854212	1.61036796	0.000887	0.00488

hsa-mir-940	1.34364842	1.358806591	0.000982	0.005331
hsa-mir-573	2.87887946	-0.11444892	0.001073	0.0057
hsa-mir-196b	1.65335729	8.257383603	0.001425	0.007444
hsa-mir-3652	2.22769609	0.536660377	0.001448	0.007446
hsa-mir-3677	1.12439427	2.132937115	0.001506	0.007603
hsa-mir-196a-1	1.7870544	6.394389032	0.001609	0.008059
hsa-mir-636	2.12309025	-0.47470943	0.001703	0.008461
hsa-mir-6715b	4.11616458	-0.22288496	0.001748	0.008604
hsa-mir-196a-2	1.73796057	6.597133062	0.00176	0.008604
hsa-mir-4449	3.40550792	1.116092645	0.001893	0.009105
hsa-mir-3682	1.11872208	0.733886413	0.002021	0.009648
hsa-mir-210	1.3938025	9.17027038	0.002049	0.009706
hsa-mir-550a-3	1.31588907	0.664019523	0.002363	0.011023
hsa-mir-1293	2.64598894	2.277298222	0.002422	0.011125
hsa-mir-675	2.57467817	5.515192716	0.002635	0.011928
hsa-mir-3065	-1.012894	5.763212646	0.002786	0.012518
hsa-mir-612	3.46180148	-0.5274243	0.002914	0.012995
hsa-mir-584	1.28557986	6.943129604	0.00295	0.01306
hsa-mir-4745	2.67939995	-0.48496545	0.003141	0.013809
hsa-mir-1269a	3.8295416	6.523381085	0.003507	0.015092
hsa-mir-508	2.80360156	4.916667107	0.004076	0.017294
hsa-mir-1910	2.85433028	-0.1434092	0.004158	0.017519
hsa-mir-3150b	1.96742184	1.066713239	0.004283	0.017875
hsa-mir-3690-1	1.6146483	0.122107284	0.004301	0.017875
hsa-mir-99a	-1.0328006	8.377608755	0.004419	0.018038
hsa-mir-34c	2.02661921	4.504745578	0.004443	0.018038
hsa-mir-3691	1.65294432	-0.25119643	0.004534	0.018224
hsa-mir-516a-1	4.98036342	2.146565988	0.006127	0.023544
hsa-mir-512-1	5.47613305	2.123773208	0.006473	0.024564
hsa-mir-1228	1.1576812	0.365698587	0.007031	0.026354
hsa-mir-5003	1.62623599	-0.39866387	0.007146	0.026623
hsa-mir-504	-1.1552111	0.096742579	0.007394	0.027214
hsa-mir-183	1.06198543	12.71113259	0.007639	0.027522
hsa-mir-6854	1.18749638	1.081503058	0.007692	0.027522
hsa-mir-4661	1.18943795	2.499712181	0.007703	0.027522
hsa-mir-526b	4.86212544	3.072364985	0.007851	0.02762
hsa-mir-1911	4.31584668	1.993167864	0.007866	0.02762
hsa-mir-944	2.75484624	6.953768088	0.00812	0.028189
hsa-mir-373	9.1745082	3.917918497	0.008753	0.030216
hsa-mir-4517	2.18159727	-0.46630372	0.009004	0.030908
hsa-mir-518c	5.12311756	1.846559033	0.0094	0.032086
hsa-mir-520d	5.11084451	0.38896586	0.009663	0.03244
hsa-mir-519a-1	4.47025436	1.988333322	0.010504	0.034692
hsa-mir-518a-2	5.59983994	0.741852655	0.011042	0.036271

hsa-mir-522	5.09732148	1.029912636	0.011206	0.036613
hsa-mir-7-3	1.64341497	2.256686094	0.011469	0.037275
hsa-mir-605	-1.1107749	-0.5322416	0.011785	0.0381
hsa-mir-512-2	5.46712458	2.125892709	0.012635	0.040453
hsa-mir-3176	1.68481869	-0.49697443	0.012646	0.040453
hsa-mir-516a-2	4.92116662	1.956611656	0.013008	0.041394
hsa-mir-205	2.51130639	12.67824798	0.013202	0.041796
hsa-mir-520f	5.66393146	2.000501062	0.013559	0.042704
hsa-mir-509-2	2.75165162	2.099483926	0.013645	0.042754
hsa-mir-6783	1.32834292	-0.30649178	0.014026	0.043725
hsa-mir-520a	4.74145557	2.877839597	0.015413	0.047563
hsa-mir-3941	1.08751312	-0.02168971	0.015816	0.048317
